# A systematic review and meta-analysis of Penner serotype prevalence of *Campylobacter jejuni* in low- and middle-income countries

**DOI:** 10.1371/journal.pone.0251039

**Published:** 2021-05-05

**Authors:** Tegan N. Clarke, Megan A. Schilling, Luca A. Melendez, Sandra D. Isidean, Chad K. Porter, Frédéric M. Poly

**Affiliations:** 1 General Dynamics Information Technology, Silver Spring, MD, United States of America; 2 Enteric Diseases Department, Infectious Diseases Directorate, Naval Medical Research Center, Silver Spring, MD, United States of America; 3 George Washington University School of Public Health, Washington, DC, United States of America; 4 Henry M. Jackson Foundation, Bethesda, MD, United States of America; USDA-ARS Eastern Regional Research Center, UNITED STATES

## Abstract

**Introduction:**

While *Campylobacter jejuni* is a leading foodborne bacterial pathogen worldwide, it poses a particular risk to susceptible populations in low- and middle-income countries (LMICs). A capsule-conjugate vaccine approach has been proposed as a potential solution, but little information exists on circulating *C*. *jejuni* capsule types in LMICs. The capsule is the major serodeterminant of the Penner typing scheme, which is based on serum recognition of *Campylobacter* heat-stable antigens. We conducted a systematic review and meta-analysis to estimate the distribution of Penner serotypes associated with *C*. *jejuni* enteritis in LMICs. Vaccine coverage assessments for hypothetical regional and global *C*. *jejuni* vaccines were also estimated.

**Methods:**

A systematic review of the literature published from 1980 to 2019 was performed using PubMed, Scopus, and Web of Science databases. Articles were assessed for eligibility and data were abstracted. Pooled *C*. *jejuni* serotype prevalence in LMICs was estimated by region and globally using random-effects models.

**Results:**

A total of 36 studies were included, capturing 4,434 isolates from LMICs. Fifteen serotypes were present in a sufficient number of studies to be included in analyses. Among these, HS4c was the most common serotype globally (12.6%), though leading capsule types varied among regions. HS2, HS3c, HS4c, HS5/31, HS8/17, and HS10 were all among the 10 most common region-specific serotypes.

**Conclusions:**

The results of this review suggest that an octavalent vaccine could provide up to 66.9% coverage of typable strains worldwide, and 56.8–69.0% regionally. This review also highlights the paucity of available data on capsules in LMICs; more testing is needed to inform vaccine development efforts.

## Introduction

Diarrheal disease accounts for 1 in 9 child deaths and is among the leading causes of death among children under five years of age [1, 2]. *Campylobacter jejuni* is a common foodborne pathogen and one of the leading causes of infectious diarrhea worldwide [3]. *C*. *jejuni* is also associated with Guillain-Barré Syndrome (GBS), a rare but potentially life-threatening autoimmune condition, as well as less severe but more common long-term sequelae, including reactive arthritis and irritable bowel syndrome [4–6].

While largely occurring as sporadic infections and outbreaks in high-income countries (HICs) [7], *C*. *jejuni* infection is endemic in low- and middle-income countries (LMICs) [8–11]. In the Malnutrition and Enteric Disease (MAL-ED) birth cohort study, *Campylobacter* spp. were among the three most common bacterial causes of diarrheal disease in infants from birth through the second year of life [12, 13]. Up to 85% of children were infected by one year of age, with one-third of those cases resulting in persistent infection [14]. In the Global Enteric Multicenter Study (GEMS), *Campylobacter* was a leading pathogen associated with moderate-to-severe diarrhea in children aged ≤5 years in Pakistan, Bangladesh, and India [15, 16]. In addition to its association with malnutrition, *Campylobacter* infection is linked to inflammation and growth deficits [16–21], all of which have implications for long-term health, education, and economic prospects [22–26].

*Campylobacter* antibiotic resistance is a growing concern among public health agencies, and few alternatives exist to treat multidrug-resistant *C*. *jejuni*. Fluoroquinolones, once first-line interventions for suspected campylobacteriosis, have been rendered increasingly ineffective by antimicrobial use in industrial agriculture [27, 28]. The World Health Organization and Centers for Disease Control and Prevention have both identified drug-resistant *Campylobacter* as a serious threat to global health [29, 30]. The need for alternatives to antimicrobials highlights the need for increased vaccine development efforts and other methods of prevention.

Though several candidates have been evaluated over the last 20 years, there are currently no licensed human vaccines against *C*. *jejuni* [31]. An increased understanding of the role of the *C*. *jejuni* capsular polysaccharide (CPS) in virulence [32] has paved the way for a conjugated polysaccharide vaccine. To date, monovalent prototypes have demonstrated efficacy in non-human primate models [33]; however, prioritization of the capsule targets for a final multivalent formulation is hampered by a lack of data on *C*. *jejuni* capsule types circulating in LMICs, where disease burden is highest [8–10, 34].

### Penner serotyping

CPS is the major serodeterminant of the Penner typing scheme [35, 36]. Penner serotyping uses passive hemagglutination (PHA) to classify *C*. *jejuni* based on heat-stable (HS) antigens [37, 38]. High variability in the *C*. *jejuni* genome results in a variety of CPS moieties across serotypes [32, 39]. There are currently 47 recognized *C*. *jejuni* Penner serotypes, which, due to cross-reactivity of related CPS structures, can be organized into 35 CPS types [40].

For over 30 years, the Penner PHA serotyping scheme was considered the gold standard for serotyping *C*. *jejuni*, but the cost and complexity of maintaining a serum reference library limited its reach [41]. Beginning in the late 1990s, some laboratories serotyped using a commercially available antisera kit of 25 CPS types, manufactured by Denka Seiken of Japan [42]. A multiplex PCR (mPCR) method for capsule characterization was later developed in 2010 [43] and has since largely replaced antisera-based methods of Penner typing.

Identifying the most prevalent *C*. *jejuni* capsule types is essential for the development of a broadly effective capsule-conjugate vaccine. The goal of this systematic review and meta-analysis was to estimate the prevalence and geographic variability of *C*. *jejuni* capsule types among clinical isolates in LMICs. Pooled estimates for coverage of hypothetical regional and global vaccines with increasing valency were also calculated.

## Methods

### Search strategy

This review was conducted according to the Preferred Reporting Items for Systematic Reviews and Meta-Analyses (PRISMA) Statement [44, 45]. Relevant studies were identified through a literature search of PubMed, Scopus, and Web of Science databases. Search queries for each database were developed in consultation with a knowledge synthesis librarian, and can be found in the S1 Text. Citations and reference lists of relevant articles were hand-searched, and experts in *C*. *jejuni* research were consulted to identify studies not captured by the database searches.

### Study screening

Two reviewers independently screened studies by title and abstract for inclusion. Discrepancies in eligibility assessments were discussed; disagreements were arbitrated by a third reviewer. Inclusion was limited to English-language studies published between 1980 and 2019 with Penner-typed *C*. *jejuni* human fecal isolates. Review articles, studies containing fewer than 10 isolates, isolates from animal or environmental sources, and studies with apparent selection biases (e.g., studies examining one specific serotype) were excluded.

### Data abstraction and analysis

Data were abstracted by two independent reviewers; disagreements were arbitrated by a third reviewer. Isolates reported as more than one HS type not known to commonly complex were counted as reacting to each listed serotype. For example, an isolate reported as belonging to serotype HS19/42 would be counted as being both an HS19 and an HS42 isolate. The HS3 complex (HS3c) was defined as including HS3, 13, and 50. The HS4 complex (HS4c) was defined as including HS4, 13, 16, 43, 50, 62, 63, 64, and 65. Isolates from outbreaks, GBS patients, and isolates of *C*. *coli* were not included in analyses.

Studies were classified by country and separated into regions: Africa, North & South America (Americas), Asia, Europe, and Oceania. Based on historical data published by the World Bank, countries were classified by income level as low (LIC), low-middle and upper-middle (MIC), and high (HIC) based on the year in which sample collection started [46]. If the year of first sample collection was not specified, the year of publication was used for classification. For studies in which sample collection began before 1987, income classification in 1987 was used. Where authors’ descriptions and data allowed for distinction between populations within a study, studies were abstracted as separate observations.

Statistical analyses were performed using the stats and meta packages in R version 3.6.3 software [47, 48]. Statistical heterogeneity was assessed via Cochran’s Q test and I-squared (I^2^) statistics [49, 50]. Random effects models (REMs; DerSimonian-Laird method) [51] were employed to calculate capsule-specific pooled prevalence estimates and associated 95% confidence intervals (CI) using the transformed log of capsule proportions. Vaccine coverage assessments for hypothetical regional and global *C*. *jejuni* vaccines were estimated via the stepwise addition of the most globally prevalent CPS types identified in REMs. In accordance with PRISMA guidelines, publication bias was assessed visually and statistically using funnel plots and Egger’s test, respectively [52].

## Results

**Fig 1:**
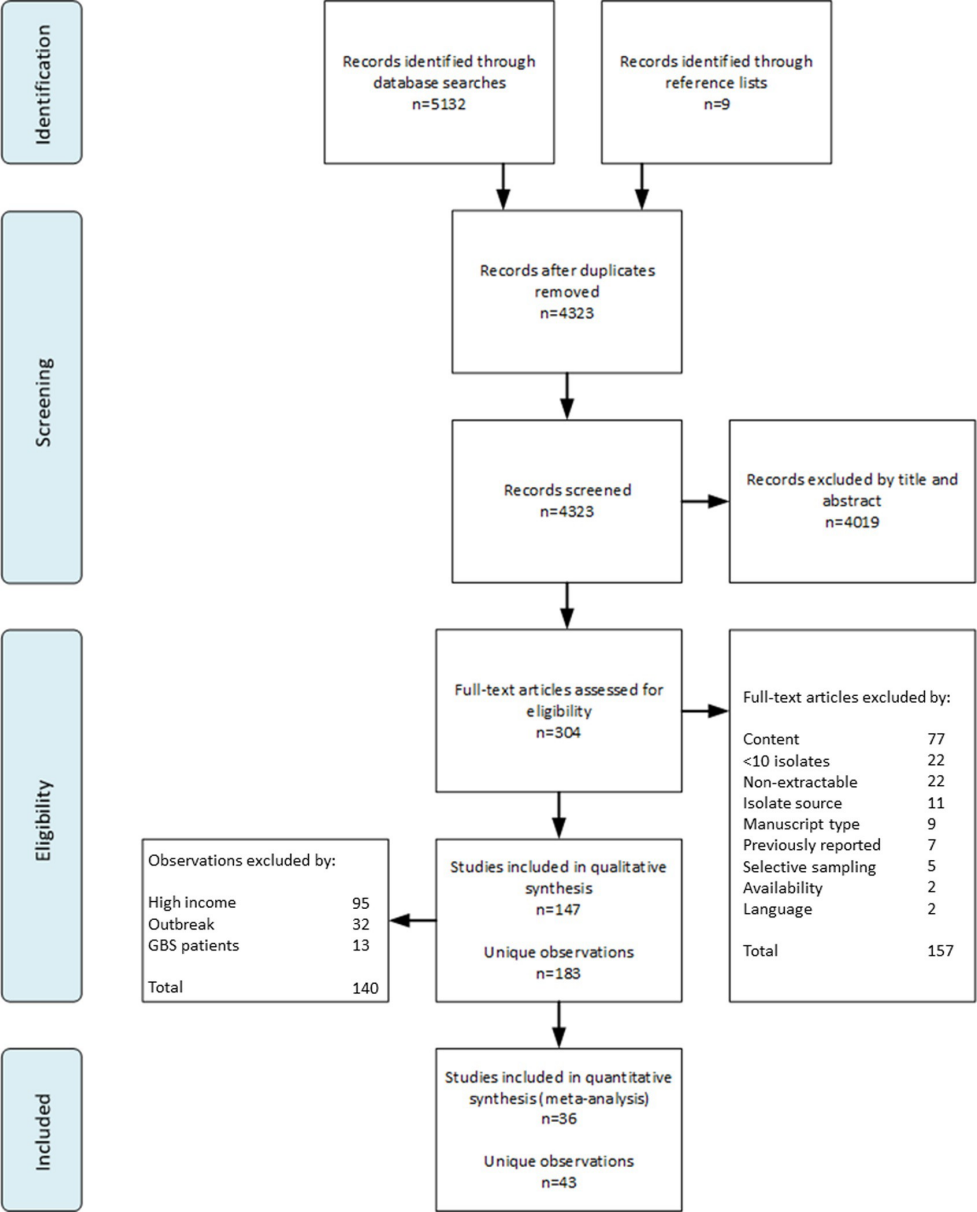
PRISMA flow diagram for identification and selection of included studies.

As shown in Fig 1, 5,132 publications were identified through database searches, and an additional 9 articles were identified through reference lists and consulting experts in the field. Of the 304 full-text articles retrieved and reviewed, a total of 157 were excluded. The most common reasons for exclusion were content (n = 77; 49.0%), non-extractable data (n = 22; 14.0%), and containing fewer than 10 isolates (n = 22; 14.0%).

From the eligible 147 studies, 183 unique observations were abstracted. These studies reported a total of 44,799 sporadic enteritis isolates collected from 46 countries between 1978 and 2015 (Tables 1 and S1). A total of 45 of these observations were excluded from analyses for containing isolates from GBS patients (n = 13; 28.9%) or outbreaks (n = 32; 71.1%). An additional 95 observations were excluded due to their collection in an HIC setting.

**Table 1:** Detailed characteristics of observations included in the meta-analysis.

Region	First Author	Year of Publication	Country	Income	n^a^	Age	Typing Method
Africa	Georges-Courbot [53]	1986	Central African Republic	Low^b^	113	Pediatric	PHA^c^
	Lastovica [54]	1986	South Africa	Middle^b^	258	Pediatric	PHA
	Lastovica [55]	1986	South Africa	Middle^b^	23	Pediatric	PHA
	Mølbak [56]	1988	Liberia	Low^b^	22	Pediatric	PHA
	Georges-Courbot [57]	1989	Central African Republic	Low^b^	209	Pediatric	PHA
	Asrat [58]	1997	Ethiopia	Low^b^	56	Mixed	PHA
	Smith [59]	1997	Nigeria	Low	29	Pediatric	PHA
	Smith [60]	1998	Nigeria	Low	20	Not Specified	PHA
	Smith [61]	2000	Nigeria	Low	29	Not Specified	PHA
	Smith [62]	2000	Nigeria	Low	21	Not Specified	PHA
	Wierzba [63]	2008	Egypt	Middle	21	Pediatric	PHA
	Sainato [64]	2017	Egypt	Middle	272	Pediatric	mPCR
Americas	Sjögren [65]	1989	Mexico	Middle^b^	136	Pediatric	PHA
	Nachamkin [66]	2007	Mexico	Middle	44	Pediatric	PHA
	Neitenbach [67]	2019	Peru	Middle	352	Pediatric	mPCR
	Rojas^d^ [68]	2019	Peru	Middle	184	Pediatric	mPCR
	Rojas^d^ [68]	2019	Peru	Middle	270	Pediatric	mPCR
Asia	Neogi [69]	1987	Bangladesh	Low^b^	102	Mixed	PHA
	Tay [70]	1995	Malaysia	Middle	26	Mixed	PHA
	Nishimura [71]	1996	China	Low	85	Not Specified	PHA
	Li [72]	2001	China	Low	90	Mixed	PHA
	Prasad [73]	2002	India	Low	23	Mixed	PHA
	Boonmar [74]	2005	Thailand	Middle	50	Pediatric	Commercial Kit
	Boonmar [75]	2007	Thailand	Middle	70	Not Specified	Commercial Kit
	Islam^e^ [76]	2009	Bangladesh	Low	39	Not Specified	PHA
	Poly^f^ [77]	2015	Thailand	Middle	263	Adult	mPCR
	Poly^f^ [77]	2015	Thailand	Middle	51	Adult	mPCR
	Poly [77]	2015	Thailand	Middle	515	Mixed	mPCR
	Poly [77]	2015	Nepal	Low	46	Adult	mPCR
	Poly [77]	2015	Nepal	Low	96	Mixed	mPCR
	Poly [77]	2015	Cambodia	Low	25	Pediatric	mPCR
	Islam [78]	2017	Bangladesh	Low	367	Pediatric	mPCR
Europe	Annan-Prah [79]	1988	Yugoslavia	Middle	55	Not Specified	PHA
	Varga [80]	1990	Hungary	Middle^b^	37	Pediatric	PHA
	Varga [81]	1998	Hungary	Middle	101	Mixed	PHA
	Steinhauserová [82]	1999	Czech Republic	Middle	88	Mixed	PHA
	Chatzipanagiotou^g^ [83]	2003	Greece	Middle	31	Pediatric	Commercial Kit
	Sonnevend [84]	2006	Hungary	Middle	92	Not Specified	Commercial Kit
	Grozdanova [85]	2011	North Macedonia	Middle	21	Not Specified	Commercial Kit
	Miljkovic-Selimovic [86]	2011	Serbia	Middle	29	Not Specified	PHA
	Trajkovska-Dokic [87]	2011	North Macedonia	Middle	26	Pediatric	Commercial Kit
	Trajkovska-Dokic^h^ [88]	2016	North Macedonia	Middle	21	Mixed	Commercial Kit
	Trajkovska-Dokic^h^ [88]	2016	North Macedonia	Middle	26	Mixed	Commercial Kit

a Total *C*. *jejuni* isolates. *C*. *coli* and other isolates are not included in this analysis.

b Income data for year of first sample collection not available until 1987; 1987 classification used.

c Passive hemagglutination technique originally described by Penner and Hennesy [37].

d Paper includes a rural (n = 184) and urban (n = 270) cohort.

e Paper includes GBS and enteritis patients; only enteritis isolates used.

f Includes both an adult military (n = 263) and adult traveler (n = 51) cohort.

g Paper includes a 1987 and 1997 cohort. Only 1987 cohort used.

h Paper includes a 2010 (n = 21) and 2015 (n = 26) cohort.

Ultimately, a total of 36 studies from LMICs were abstracted into 43 unique observations. Among these observations, the median year of publication was 2005 (Interquartile Range (IQR): 1996–2015) (Table 1). The majority of observations were from Asia (n = 15; 34.9%) and Africa (n = 12; 27.9%). Of the included observations, 19 (44.2%) included isolates sourced exclusively from pediatric populations (defined as <18 years of age), 3 (7.0%) sourced exclusively from adult populations (defined as ≥18 years of age), and 11 (25.6%) sourced from mixed-age populations. The most common typing method used was PHA (n = 24; 55.8%), followed by mPCR (n = 11; 25.6%), and the commercially available kit (n = 8; 18.6%). The mPCR method was used in 11 of the 13 (84.6%) observations published in the last 5 years.

**Fig 2:**
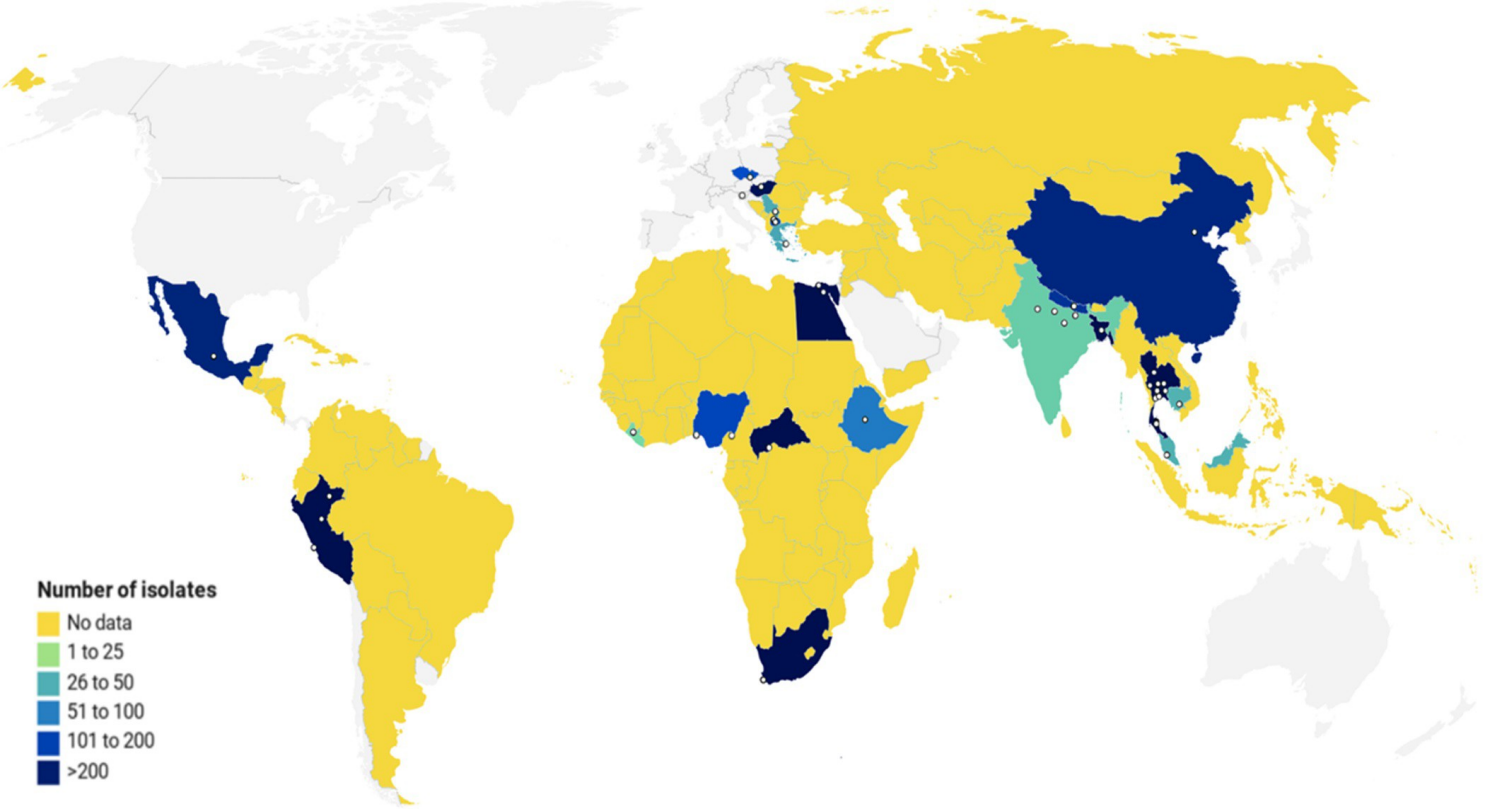
Geographic sources and number of *C*. *jejuni* isolates from included studies. The map depicts LMICs in color according to number of isolates reported. Yellow represents no data (0 isolates), while gradations of blue indicate increasing numbers of isolates. Sampling sites are represented by white dots. Visualization created with Datawrapper.

The 43 abstracted observations yielded 4,434 isolates from 21 countries (Table 1 and Fig 2). The number of isolates per observation ranged from 20 to 515, with a median of 51 isolates (IQR: 26–113). Thailand and Peru yielded the largest numbers of isolates (949 and 806, respectively). Liberia, India, and Cambodia yielded the fewest isolates (22, 23, and 25, respectively). The number of unique countries represented in each region were: Africa, 6; Americas, 2; Asia, 7; and Europe, 6. No isolates were found from LMICs in Oceania.

Forest plots were constructed to graphically summarize capsule-specific meta-analyses (S1–S15 Figs). The most prevalent capsule type based on pooled global estimates was HS4c, which accounted for 12.6% (95% Confidence Interval (CI): 10.2%-15.6%) of typable *C*. *jejuni* isolates (Table 2). Also common globally were HS2 (12.4%; 95% CI: 9.4%-16.1%), HS5/31 (9.9%; 95% CI: 7.9%-12.3%), and HS3c (9.5%; 95% CI: 7.4%-12.1%). Several capsule types appeared to exhibit some geographic variability. For example, the prevalence of HS2 in the Americas (6.6%; 95% CI: 5.1%-8.5%) was found to be lower than other regions (Africa: 11.1%, 95% CI: 8.9%-13.9%; Asia: 14.0%, 95% CI: 8.7%-21.8%; Europe: 18.5%, 95% CI: 11.2%-29.0%). In Europe, capsule types HS1/44 (13.8%; 95% CI: 8.7%-21.3%) and HS8/17 (14.8%; 95% CI: 10.1%-21.3%) were more prevalent than in Africa (HS1/44: 8.1%, 95% CI: 6.0%-10.9%; HS8/17: 6.1%, 95% CI: 1.8%-19.0%), the Americas (HS1/44: 3.5%, 95% CI: 1.5%-8.1%; HS8/17: 6.7%, 95% CI: 5.2%-8.6%), and Asia (HS1/44: 5.5%, 95% CI: 4.1%-7.4%; HS8/17: 8.3%, 95% CI: 6.0%-11.4%).

**Table 2:** Global and regional pooled prevalence (95% confidence intervals) of 15 *C*. *jejuni* capsule types among all typable *C*. *jejuni*.

	Global	Africa	Asia	Americas	Europe
**HS1/44**	7.1 (5.4, 9.4)	8.1 (6.0, 10.9)	5.5 (4.1, 7.4)	3.5 (1.5, 8.1)	13.8 (8.7, 21.3)
**HS2**	12.4 (9.4, 16.1)	11.1 (8.9, 13.9)	14.0 (8.7, 21.8)	6.6 (5.1, 8.5)	18.5 (11.2, 29.0)
**HS3c**	9.5 (7.4, 12.1)	14.7 (11.7, 18.3)	9.7 (6.8, 13.6)	7.7 (4.5, 12.9)	6.7 (3.4, 12.8)
**HS4c**	12.6 (10.2, 15.6)	11.3 (7.3, 17.2)	11.0 (7.7, 15.4)	14.5 (12.1, 17.3)	16.5 (9.5, 27.1)
**HS5/31**	9.9 (7.9, 12.3)	11.5 (7.6, 17.1)	10.6 (7.2, 15.2)	7.7 (5.0, 11.7)	8.5 (5.3, 13.4)
**HS6/7**	5.3 (3.5, 7.9)	7.1 (4.4, 11.1)	5.9 (2.6, 12.6)	3.9 (2.3, 6.6)	6.7 (3.0, 14.4)
**HS8/17**	8.0 (5.8, 10.9)	6.1 (1.8, 19.0)	8.3 (6.0, 11.4)	6.7 (5.2, 8.6)	14.8 (10.1, 21.3)
**HS9**	3.1 (1.7, 5.4)	1.7 (0.6, 4.4)	3.3 (2.4, 4.6)	1.0 (0.2, 5.4)	6.7 (2.0, 20.3)
**HS10**	4.5 (3.5, 5.8)	5.9 (3.3, 10.2)	3.5 (2.5, 4.9)	5.7 (4.4, 7.5)	6.0 (2.7, 12.5)
**HS15**	5.5 (3.6, 8.1)	10.4 (7.0, 15.2)	3.8 (1.8, 7.8)	8.2 (6.4, 10.5)	6.6 (3.5, 12.2)
**HS19**	5.2 (2.3, 11.7)	3.4 (1.9, 6.1)	12.2 (2.3, 45.4)	5.5 (0.1, 81.4)	7.6 (2.5, 20.8)
**HS23/36**	5.8 (4.1, 8.2)	6.4 (3.6, 11.2)	7.5 (4.3, 12.9)	4.0 (2.2, 7.1)	3.4 (1.5, 7.3)
**HS37**	3.5 (2.2, 5.5)	4.3 (1.3, 13.1)	2.8 (1.9, 4.3)	2.6 (0.9, 6.9)	6.5 (3.4, 12.0)
**HS41**	4.9 (3.9, 6.2)	3.3 (2.1, 5.4)	4.6 (2.5, 8.3)	6.0 (4.5, 8.0)	6.5 (1.6, 22.4)
**HS53**	6.0 (3.8, 9.4)	9.3 (5.1, 16.2)	6.9 (3.2, 14.1)	3.8 (2.2, 6.4)	2.1 (0.5, 8.1)

Five capsule types were associated with a pooled prevalence >10% in Africa (HS3c, HS5/31, HS4c, HS2, and HS15); four capsule types achieved >10% in Asia (HS2, HS19, HS4c, and HS5/31) and Europe (HS2, HS4c, HS8/17, and HS1/44). Only 1 capsule type reached this threshold in the Americas (HS4c).

**Fig 3:**
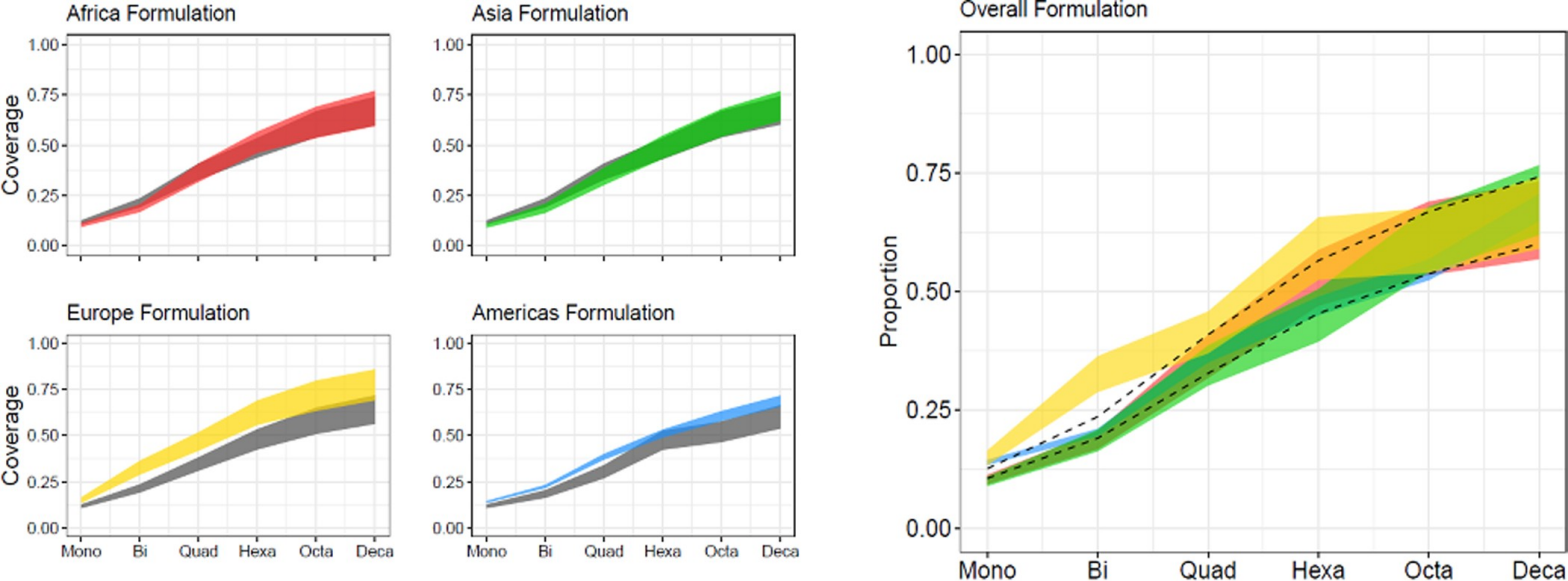
Estimates of the proportion of *C*. *jejuni* isolate coverage for a capsule-conjugate-based vaccine by region and valency using (a) region-specific and (b) global formulations. (a) The x-axis represents the coverage by a stepwise addition of the most prevalent capsule types in each region (mono = monovalent, bi = bivalent, quad = quadrivalent, hexa = hexavalent, octa = octavalent, deca = decavalent) and the y-axis represents the estimated coverage calculated by the pooled prevalence of the proposed vaccine. The shaded region shows the estimated coverage, with the lower bound including non-typable isolates in the pooled prevalence calculations and the upper bound excluding non-typable isolates. The colored area (red = Africa, green = Asia, yellow = Europe, and blue = Americas) represents the coverage achieved by a region-specific formulation (S2 Table). The gray area represents the global coverage with the use of each respective region-specific formula. (b) The x-axis represents the coverage by a stepwise addition of the most prevalent capsule types globally (mono = monovalent, bi = bivalent, quad = quadrivalent, hexa = hexavalent, octa = octavalent, deca = decavalent) and the y-axis represents the estimated coverage calculated by the pooled prevalence of the proposed vaccine. The dotted lines show the estimated coverage of the global vaccine formulation if applied to all regions (globally). The colored regions (red = Africa, blue = Americas, green = Asia, and yellow = Europe) show the estimated coverage of the global vaccine formulation in each individual region (S2 Table). The lower bound represents the estimated coverage including non-typable isolates in the pooled prevalence calculations and the upper bound represents the estimated coverage excluding non-typable isolates.

Pooled estimates for coverage of hypothetical regional and global vaccines with increasing valency were calculated (Fig 3 and S2 Table). A monovalent vaccine targeting HS4c, the most prevalent capsule type globally, yielded a point estimate of 10.6% to 12.6% when including and excluding non-typable isolates in the denominators of proportions, respectively. While a bivalent vaccine (including HS4c and HS2) would be anticipated to only cover 23.6% of typable strains globally, coverage may be as high as 36.4% in Europe. Increasing valency to a hexavalent vaccine would increase global coverage to 56.6%, with higher coverage in Europe (65.7%; 95% CI: 52.3%-77.0%) than in other geographic regions (Africa: 58.8%, 95% CI: 47.7%-69.0%; Americas: 48.9%, 95% CI: 42.8%-55.0%; Asia: 50.5%, 95% CI: 36.5%-64.5%). An additional 4 capsule types, yielding a decavalent vaccine, could cover up to 74.2% of the typable strains in LMICs.

Publication bias appeared to be present for both HS4c and HS5/31 (respective p-values <0.01; S16 Fig). While assessed per PRISMA guidelines, the relevance of these outcomes is questionable in the context of this review.

Studies using mPCR to identify isolates reported a significantly (p-value = 0.04) lower proportion of non-typable isolates (4.0%; 95% CI: 1.1%-11.9%) than studies using the commercial kit (18.0%; 95% CI: 6.8%-40.3%). Studies using traditional PHA also had lower proportions of non-typable isolates (14.0%; 95% CI: 8.5%-22.6%) than those using the commercial kit, but had higher proportions when compared to those using mPCR; however, this finding was not statistically significant (p-value = 0.50).

## Discussion

Accurate estimates of circulating *C*. *jejuni* CPS serotypes are critical to the development of a capsule-based vaccine approach to campylobacteriosis prevention. This review presents updated estimates of circulating CPS types across LMICs, providing some guidance to vaccine developers on CPS targets for a multivalent vaccine. Further, this review has illustrated that a broadly protective vaccine for *C*. *jejuni* may require fewer capsule types than current pneumococcal vaccines, which have been highly successful worldwide [89].

Notably, in spite of the differences in CPS prevalence across each of the studied regions, the most prevalent serotypes globally appeared consistently among the leading CPS types in each region. A vaccine consisting of the 10 most prevalent CPS types globally (HS1/44, HS2, HS3c, HS4c, HS5/31, HS8/17, HS10, HS15, HS23/36, and HS53) would be expected to cover almost 75% of typable strains globally, and between 70.6%-76.7% of strains between regions (S2 Table).

The generalizability of findings from this review is subject to certain limitations. First, the scope of this study is limited by the scarcity of available data from LMICs. As underscored by the map shown in Fig 2, the geographic sources of isolates in this study are few and diffuse, and most LMICs worldwide have no reported data at all. This scarcity makes it difficult to draw conclusions about variability within continents, let alone countries or regions therein. As demonstrated by Rojas et al., there may be high degrees of variability in CPS prevalence between different environments in the same country [68]. Regrettably, we were unable to expand on this observation due to the limited number of studies from any given country, and further studies on within-region serotype variability are needed to assess the consistency of this observation.

High prevalence of non-typable strains has been a major hindrance in determining valency requirements and coverage estimates for a *C*. *jejuni* CPS-based vaccine. The proportion of non-typable strains among studies included in this review ranged from 0.0% to 63.6%, with a median of 19.2% (IQR: 9.1%-33.1%). This heterogeneity may in part be attributable to differences in typing methods and changing techniques. Additionally, while some of these non-typable isolates may reflect yet-undiscovered CPS types, the phase variable nature of CPS expression in *C*. *jejuni* [32] poses a challenge in phenotypic typing methods such as PHA.

Estimating the prevalence of *C*. *jejuni* serotypes in LMICs is further complicated by a lack of standardization in methodology and reporting. The polyclonal rabbit sera used in the original PHA method are generally produced within or shared between individual laboratories, and no standard screening panel exists [34, 90]. Unfortunately, many studies included in this review did not specify whether unnamed capsule types were screened and not found (i.e., 0) or were not assessed at all (i.e., missing), which may affect prevalence and vaccine coverage estimates. Some laboratories have developed their own modifications to the Penner method [77]—ostensibly typing the same heat-stable antigens, in varying degrees of concordance with traditional PHA typing [91–93]. Further, the most readily available commercial typing kit for many laboratories, the Denka Seiken *Campylobacter* antisera kit, had the highest proportion of non-typable isolates of the methods reviewed here. The high proportion of non-typable strains may reflect isolates potentially typable by a broader screening array [42, 94].

The results of this review differ from the conclusions of an earlier systematic review conducted by Pike et al. [34]. This is not unexpected, as the number of typed isolates from LMICs (n = 4,434) has almost quadrupled since its publication (n = 1,222), largely as a consequence of the development of the mPCR typing method [43, 77]. While HS4c and HS2 remain the most common capsule types across LMICs globally, we found their prevalence (12.6% and 12.4%, respectively) to be lower than estimates reported by Pike et al. (17.5% and 16.5%, respectively). In that review, Pike et al. reported that three capsule types (HS1/44, HS2, and HS4c) covered 43.0% of LMIC isolates; the same capsule types were found in this review to cover only 32.1% of LMIC isolates. Further comparisons between the current and previous review are limited, as the previous review contained no studies from South America, and data on LMICs were not reported by region.

Conjugate polysaccharide vaccines have proven to be highly effective against mucosal pathogens, but their development remains an expensive endeavor. Inclusion of a serotype in a vaccine formulation should be warranted by epidemiologic and disease morbidity data. Some *C*. *jejuni* CPS types are well-represented in LMICs and HICs alike, and almost certainly warrant inclusion in vaccines. However, additional studies reporting CPS typing of disease-associated *C*. *jejuni* in LMICs are needed to inform formulation requirements.

Though not addressed in this review, *Campylobacter* causes substantial morbidity in industrialized nations. In the United States (US) alone, there are an estimated 1.1 million *Campylobacter*-related illnesses annually, resulting in an estimated $2.3 billion in productivity losses each year [95, 96]. Of increasing concern is the rising rate of multidrug-resistant *Campylobacter* internationally [29, 30]. In the US, strains isolated from international travelers are more likely to be resistant to at least one frontline antibiotic, limiting available treatments and highlighting the need for primary prevention among travelers to LMICs [97].

The burden of enteric infections, including *Campylobacter*, is continually evolving and requires iterative evaluations by pharmaceutical and philanthropic players interested in developing primary and secondary prevention strategies. Given that *Campylobacter* is among the most common bacterial pathogens implicated in foodborne illness worldwide, a highly effective vaccine would benefit multiple populations, including children, adult travelers, and military personnel deploying to LMICs. These data support the development of one vaccine approach that, with a combined push from government funding and private investment, could have a significant impact on global health.

## Supporting information

S1 Fig Forest plot for capsule type HS1/44. (PDF)

S2 Fig Forest plot for capsule type HS2. (PDF)

S3 Fig Forest plot for capsule type HS3c. (PDF)

S4 Fig Forest plot for capsule type HS4c. (PDF)

S5 Fig Forest plot for capsule type HS5/31. (PDF)

S6 Fig Forest plot for capsule type HS6/7. (PDF)

S7 Fig Forest plot for capsule type HS8/17. (PDF)

S8 Fig Forest plot for capsule type HS9. (PDF)

S9 Fig Forest plot for capsule type HS10. (PDF)

S10 Fig Forest plot for capsule type HS15. (PDF)

S11 Fig Forest plot for capsule type HS19. (PDF)

S12 Fig Forest plot for capsule type HS23/36. (PDF)

S13 Fig Forest plot for capsule type HS37. (PDF)

S14 Fig Forest plot for capsule type HS41. (PDF)

S15 Fig Forest plot for capsule type HS53. (PDF)

S16 Fig Funnel plots for capsule types. (PDF)

S1 Table Number of isolates (percent of total) and number of observations identified by region and income classification. (PDF)

S2 Table Pooled estimates for coverage of hypothetical regional and global vaccine formulations. Based on pooled prevalence estimates, different formulations for broadest possible coverage were assessed for each region (Af = Africa, As = Asia, E = Europe), as well as cumulatively (global). Shaded squares indicate inclusion of a capsule type in each respective formulation.
(PDF)

S1 Text Search terms. (PDF)

S1 Checklist PRISMA 2009 checklist. (DOC)

## Abbreviations

CPS: capsular polysaccharide
GBS: Guillain-Barré Syndrome
HICs: high-income countries
HS: heat-stable
LMICs: low- and middle-income countries
mPCR: multiplex PCR
PHA: passive hemagglutination
