# Supplementary figures and images for: A systematic review and meta-analysis of Penner serotype prevalence of Campylobacter jejuni in low- and middle-income countries
Source: PLoS One. 2021 May 5;16(5):e0251039. doi: 10.1371/journal.pone.0251039 (PMC8099051; doi:10.1371/journal.pone.0251039)

**S1 Fig. Forest plot for capsule type HS1/44.**

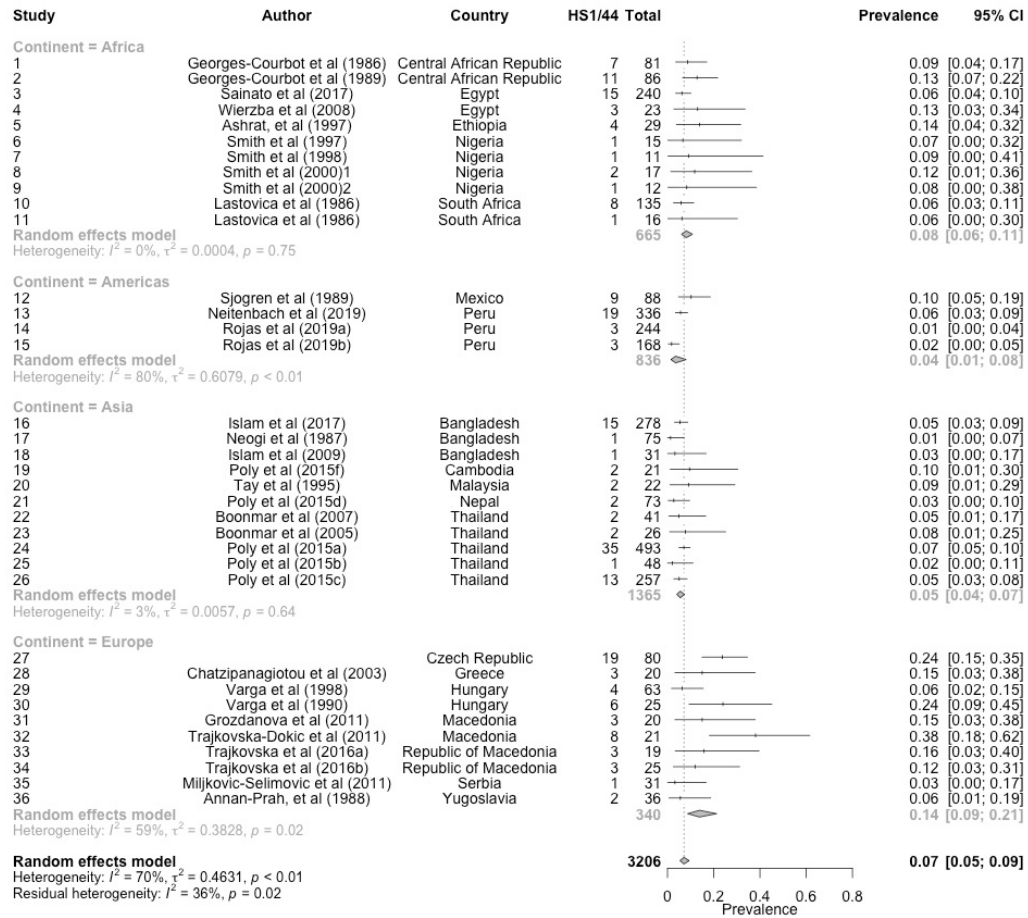

Supplement: S1 Fig — (PDF) [file pone.0251039.s001.pdf]

**S2 Fig. Forest plot for capsule type HS2.**

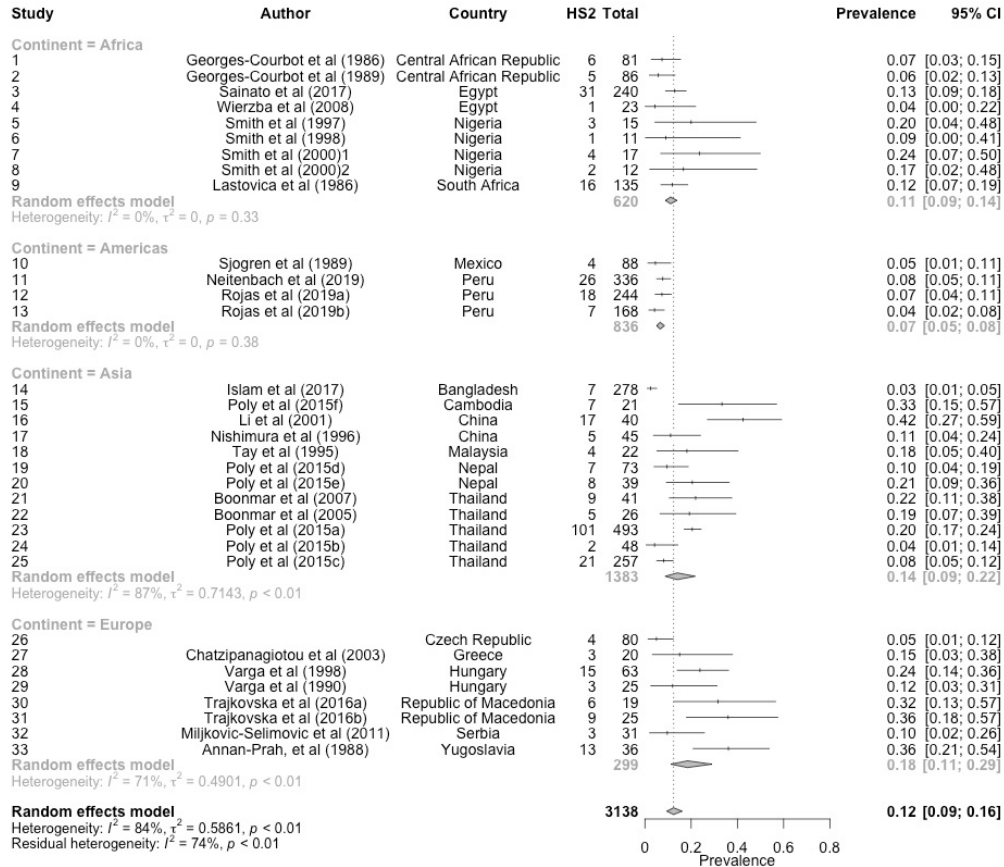

Supplement: S2 Fig — (PDF) [file pone.0251039.s002.pdf]

**S3 Fig. Forest plot for capsule type HS3c.**

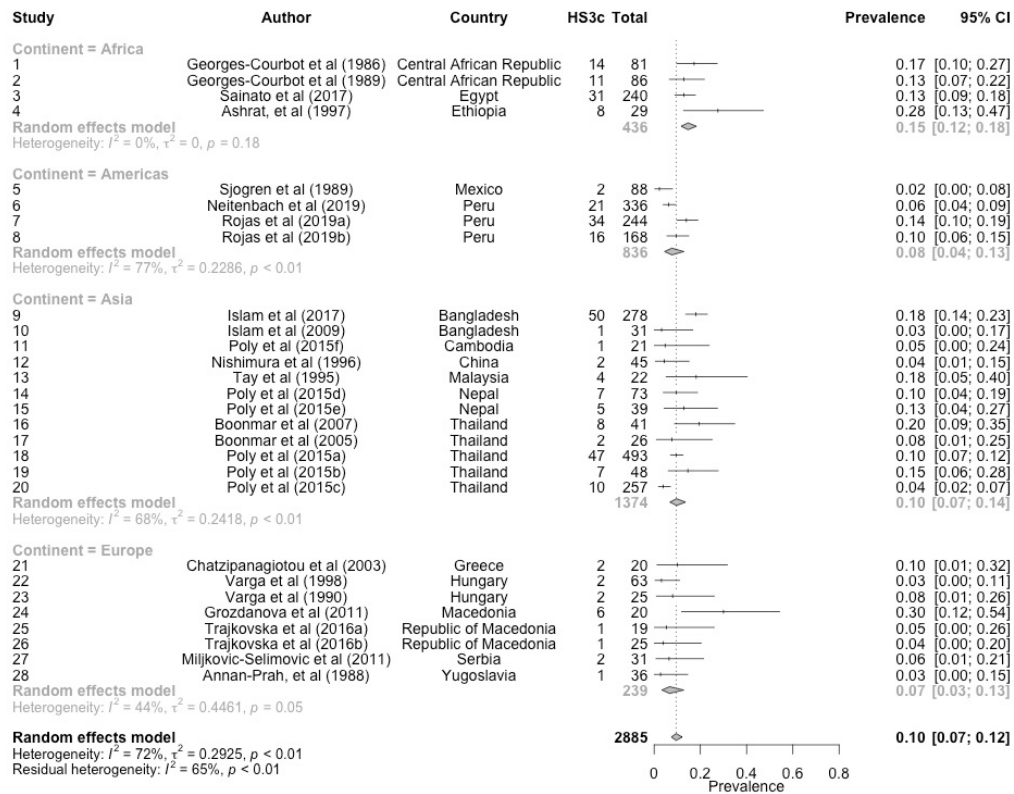

Supplement: S3 Fig — (PDF) [file pone.0251039.s003.pdf]

**S4 Fig. Forest plot for capsule type HS4c.**

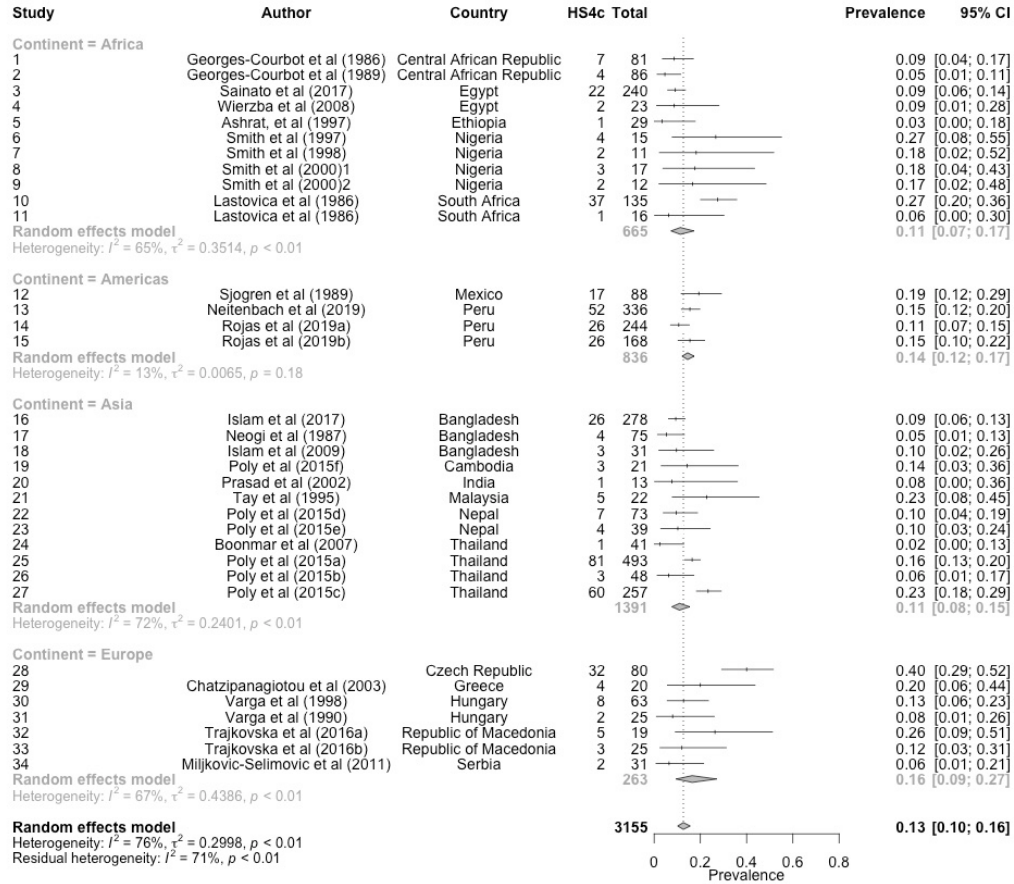

Supplement: S4 Fig — (PDF) [file pone.0251039.s004.pdf]

**S5 Fig. Forest plot for capsule type HS5/31.**

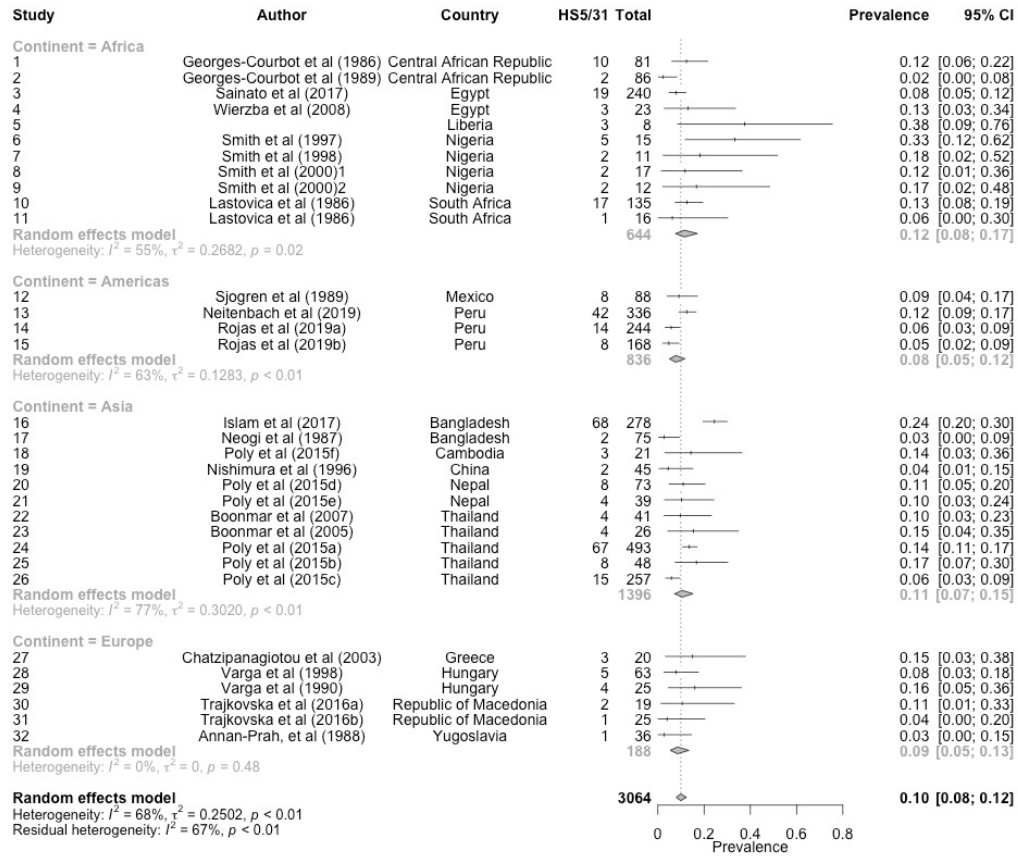

Supplement: S5 Fig — (PDF) [file pone.0251039.s005.pdf]

S6 Fig. Forest plot for capsule type HS6/7.

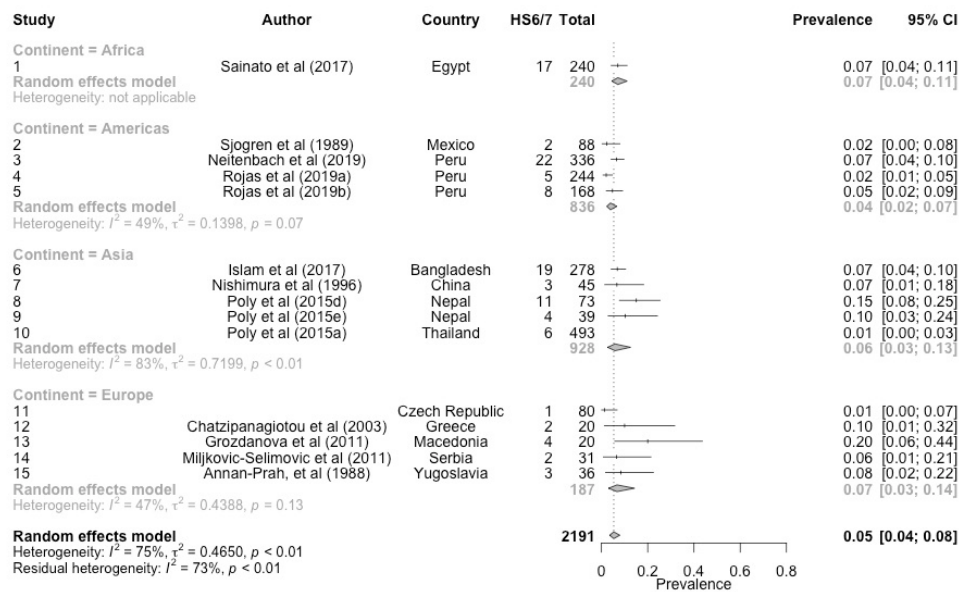

Supplement: S6 Fig — (PDF) [file pone.0251039.s006.pdf]

**S7 Fig. Forest plot for capsule type HS8/17.**

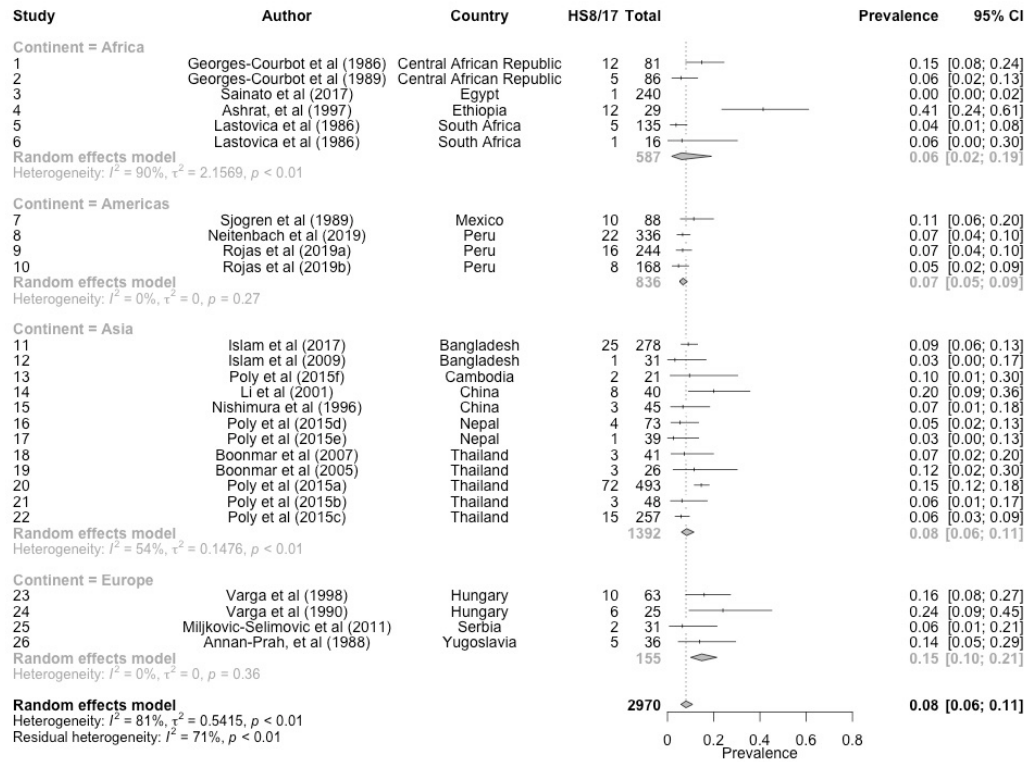

Supplement: S7 Fig — (PDF) [file pone.0251039.s007.pdf]

S8 Fig. Forest plot for capsule type HS9.

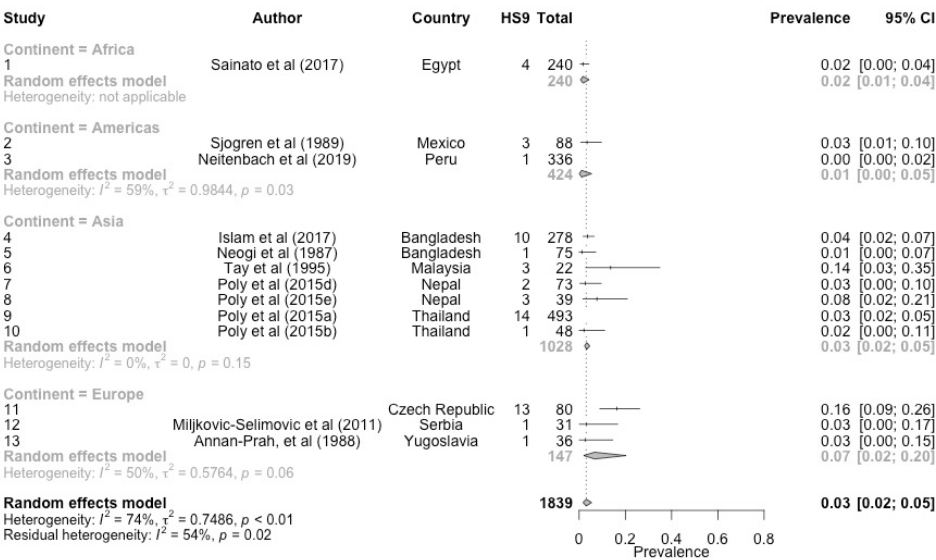

Supplement: S8 Fig — (PDF) [file pone.0251039.s008.pdf]

S9 Fig. Forest plot for capsule type HS10.

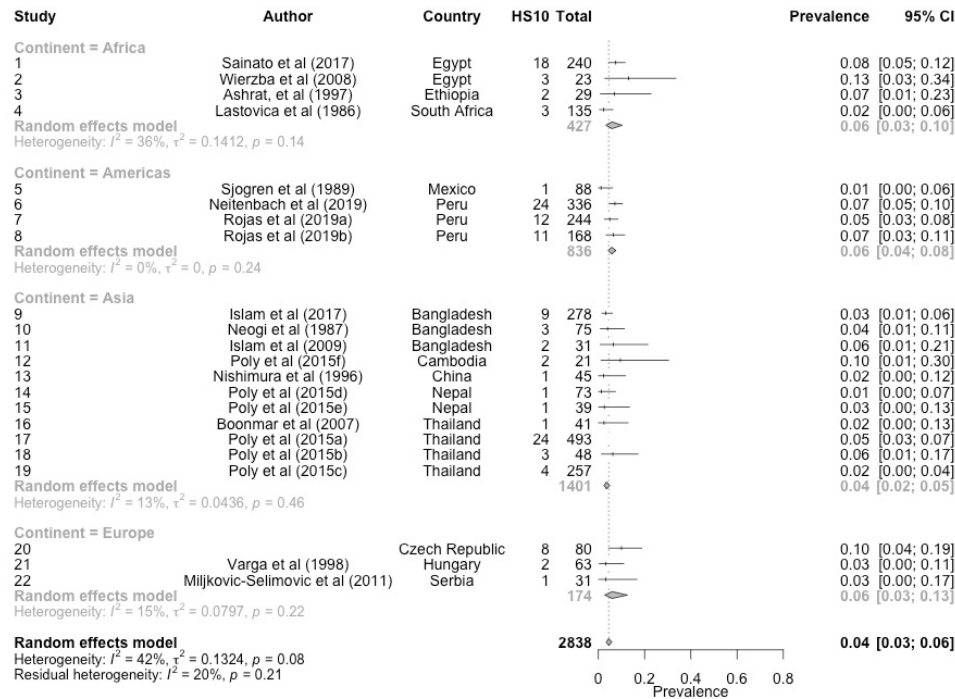

Supplement: S9 Fig — (PDF) [file pone.0251039.s009.pdf]

S10 Fig. Forest plot for capsule type HS15.

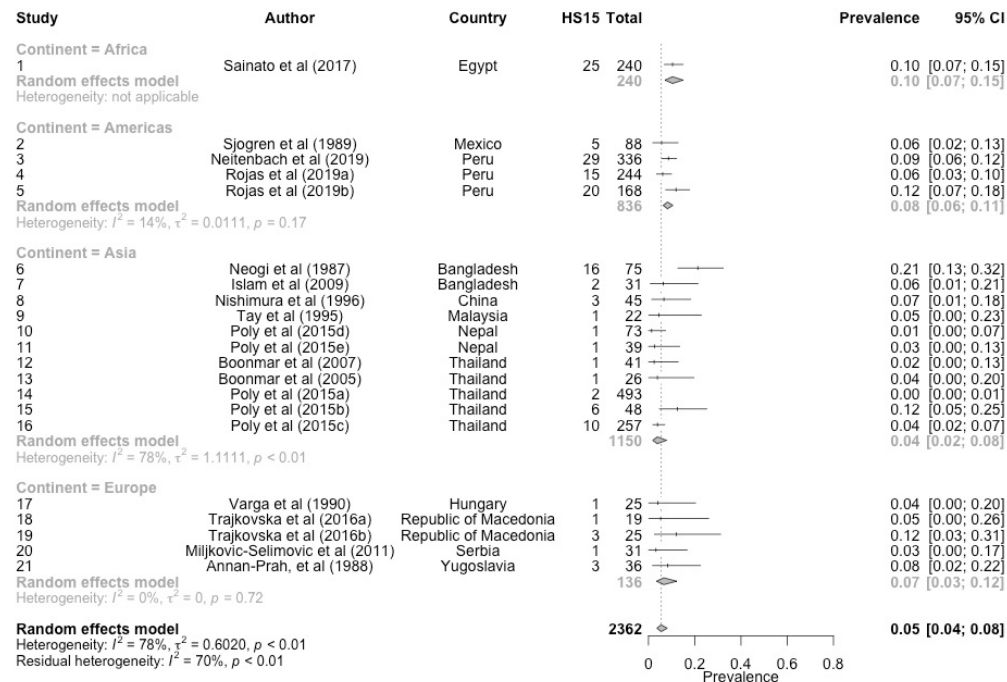

Supplement: S10 Fig — (PDF) [file pone.0251039.s010.pdf]

S11 Fig. Forest plot for capsule type HS19.

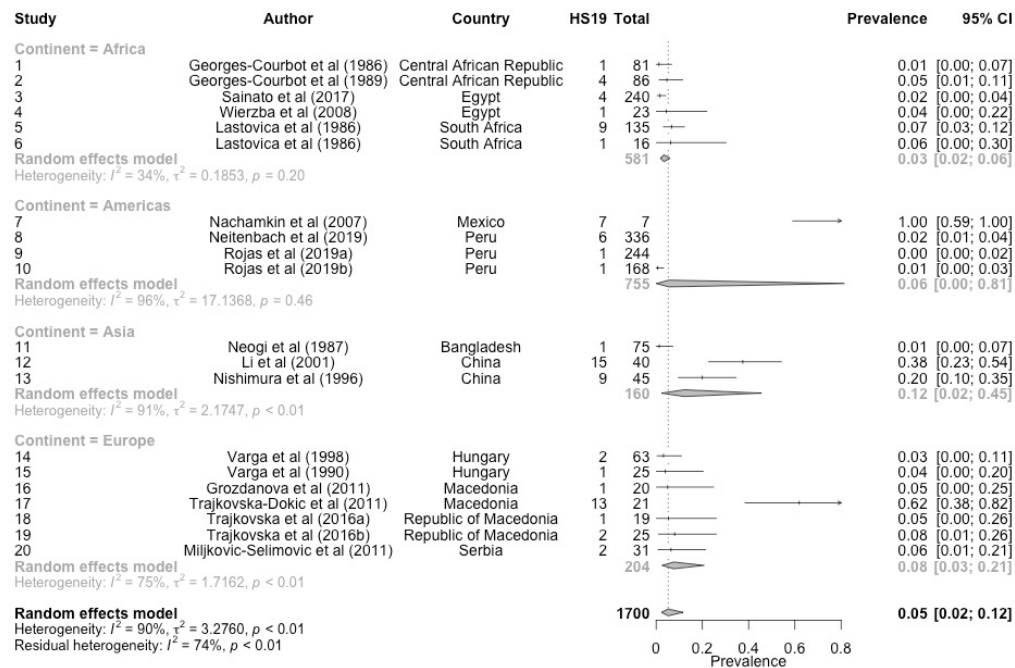

Supplement: S11 Fig — (PDF) [file pone.0251039.s011.pdf]

S12 Fig. Forest plot for capsule type HS23/36.

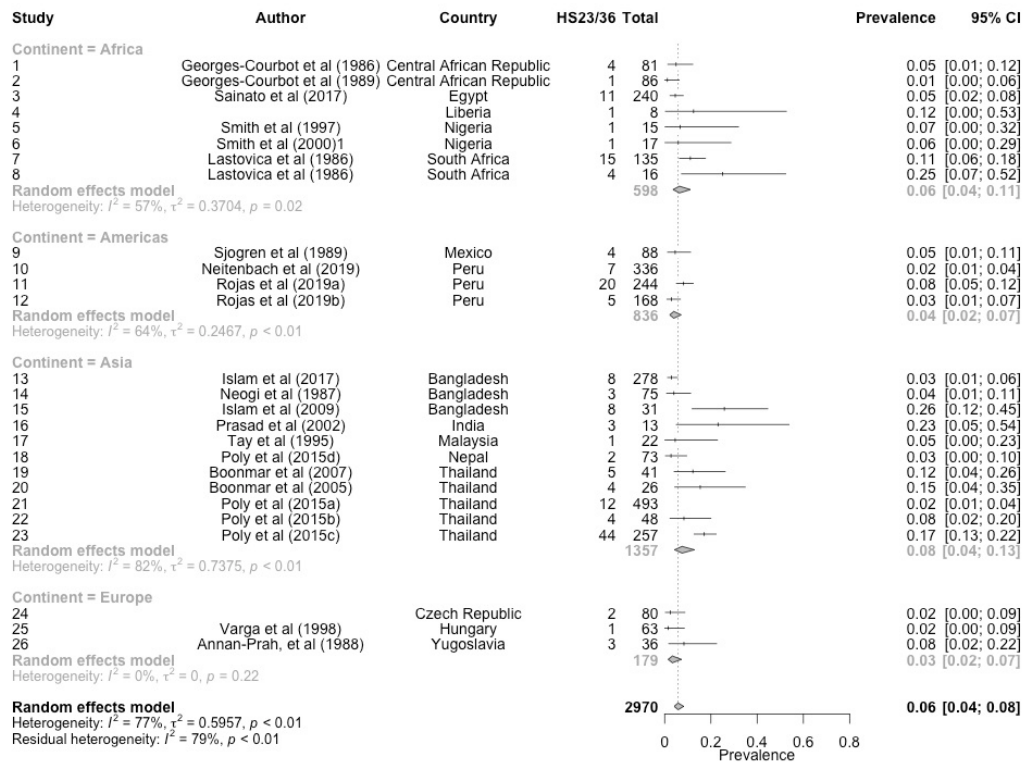

Supplement: S12 Fig — (PDF) [file pone.0251039.s012.pdf]

S13 Fig. Forest plot for capsule type HS37.

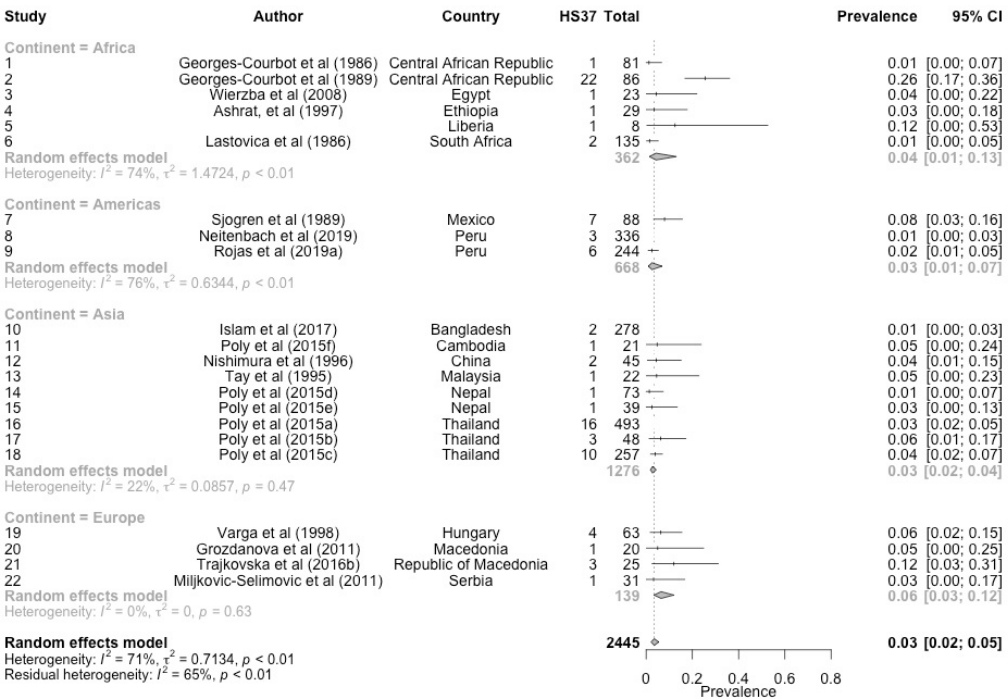

Supplement: S13 Fig — (PDF) [file pone.0251039.s013.pdf]

S14 Fig. Forest plot for capsule type HS41.

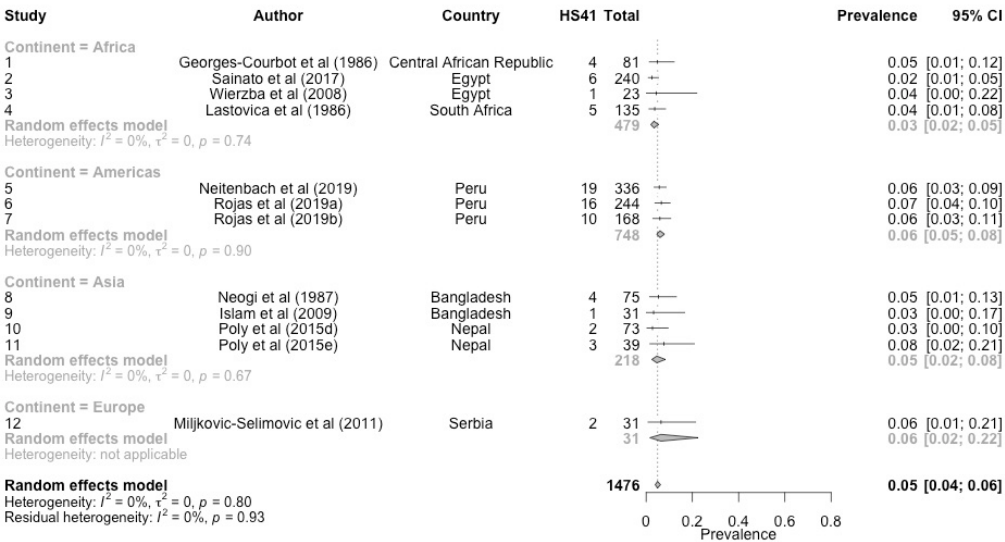

Supplement: S14 Fig — (PDF) [file pone.0251039.s014.pdf]

S15 Fig. Forest plot for capsule type HS53.

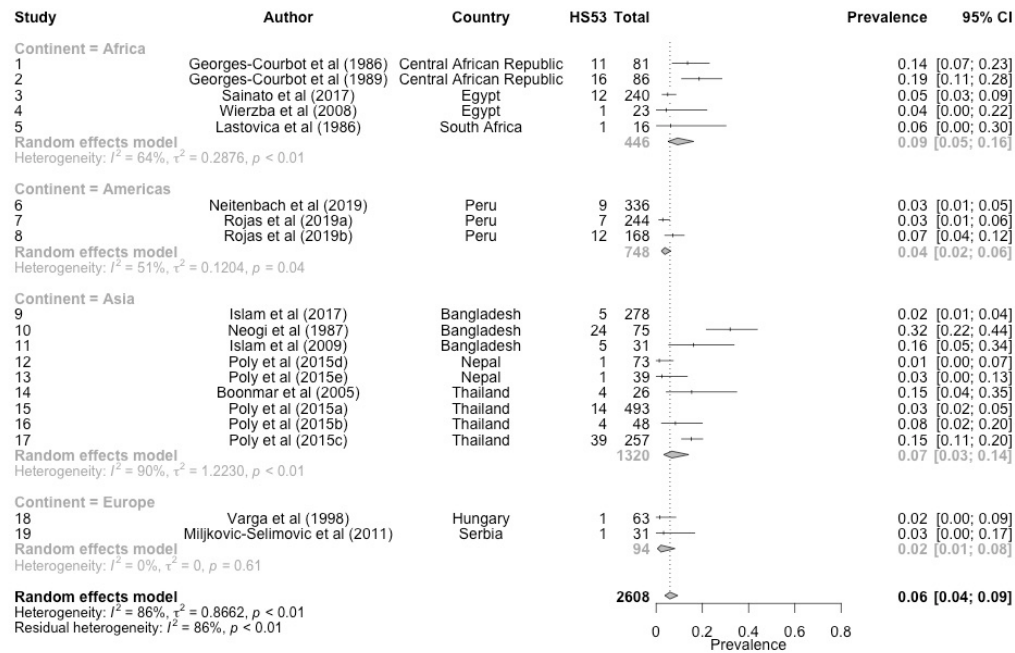

Supplement: S15 Fig — (PDF) [file pone.0251039.s015.pdf]

S16 Fig. Funnel plots for capsule types.

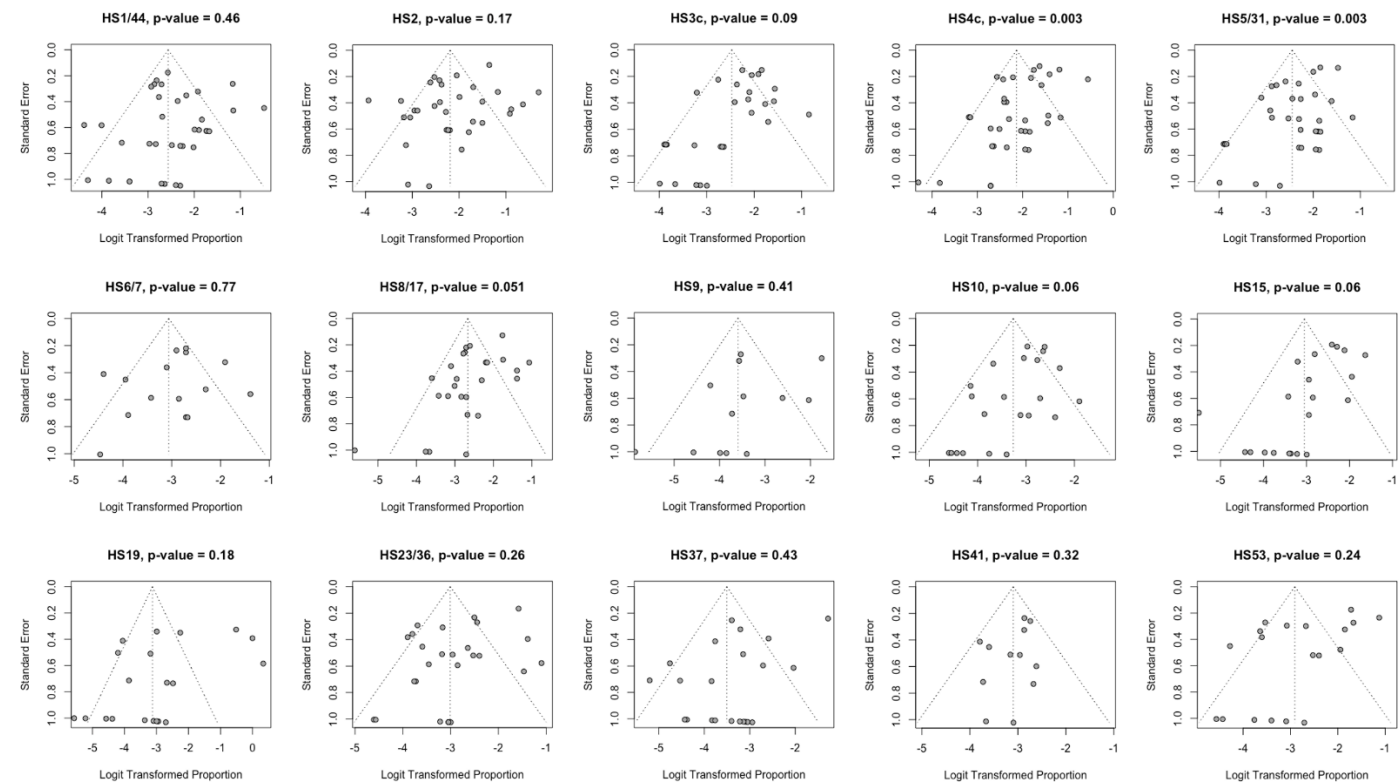

Supplement: S16 Fig — (PDF) [file pone.0251039.s016.pdf]
