# Supplementary material for: A systematic review and meta-analysis of Penner serotype prevalence of Campylobacter jejuni in low- and middle-income countries
Source: PLoS One. 2021 May 5;16(5):e0251039. doi: 10.1371/journal.pone.0251039 (PMC8099051; doi:10.1371/journal.pone.0251039)
Supplement: S1 Table — (PDF) [file pone.0251039.s017.pdf]

S1 Table. Number of isolates (percent of total) and number of observations identified by region and income classification.

			Income Classification			
			Low	Middle	High	Total
Region	Africa	Isolates (%)	499 (1.11)	574 (1.28)	0 (0)	1073 (2.40)
		Observations	8	4	0	12
	Americas	Isolates (%)	0 (0)	986 (2.20)	1510 (3.37)	2496 (5.57)
		Observations	0	5	14	19
	Asia	Isolates (%)	873 (1.95)	975 (2.18)	2318 (5.17)	4166 (9.30)
		Observations	9	6	13	28
	Europe	Isolates (%)	0 (0)	527 (1.17)	35470 (79.18)	35997 (80.35)
		Observations	0	11	57	68
	Oceania	Isolates (%)	0 (0)	0 (0)	1067 (2.38)	1067 (2.38)
		Observations	0	0	11	11
	Total	Isolates (%)	1372 (3.06)	3062 (6.83)	40365 (90.10)	44799
		Observations	17	26	95	138
