# Supplementary material for: A systematic review and meta-analysis of Penner serotype prevalence of Campylobacter jejuni in low- and middle-income countries
Source: PLoS One. 2021 May 5;16(5):e0251039. doi: 10.1371/journal.pone.0251039 (PMC8099051; doi:10.1371/journal.pone.0251039)
Supplement: S2 Table — Based on pooled prevalence estimates, different formulations for broadest possible coverage were assessed for each region (Af = Africa, As = Asia, E = Europe), as well as cumulatively (global). Shaded squares indicate inclusion of a capsule type in each respective formulation. (PDF) [file pone.0251039.s018.pdf]

S2 Table. Pooled estimates for coverage of hypothetical regional and global vaccine formulations.

		HS1/44	HS2	HS3c	HS4c	HS5/31	HS6/7	HS8/17	HS9	HS10	HS15	HS19	HS23/36	HS37	HS41	HS53	Including Non-Typable Strains					Excluding Non-Typable Strains				
																	Global	Africa	Americas	Asia	Europe	Global	Africa	Americas	Asia	Europe
Monovalent	Global				■												10.6 (8.4, 13.2)	9.3 (6.2, 13.7)	13.4 (11.1, 16.0)	8.9 (5.9, 13.4)	13.4 (7.5, 22.8)	12.6 (10.2, 15.6)	11.3 (7.3, 17.2)	14.5 (12.1, 17.3)	11.0 (7.7, 15.4)	16.5 (9.5, 27.1)
Bivalent	Global, Af, As, E		■		■												19.1 (15.4, 23.4)	16.8 (11.5, 23.8)	19.5 (16.9, 22.4)	16.3 (10.6, 24.1)	28.8 (21.2, 37.8)	23.6 (19.4, 28.4)	20.6 (13.4, 30.2)	21.1 (18.4, 23.9)	20.9 (14.7, 28.7)	36.4 (28.4, 45.2)
	Americas			■	■												16.1 (13.3, 19.4)	18.6 (16.0, 21.4)	21.5 (18.9, 24.3)	13.6 (9.0, 20.1)	16.0 (9.8, 25.1)	20.4 (17.6, 23.6)	22.4 (19.4, 25.7)	23.2 (20.5, 26.2)	18.1 (13.3, 24.3)	19.8 (12.9, 29.3)
Quadrivalent	Global, Af, As		■	■	■	■											32.8 (28.1, 37.8)	31.8 (25.1, 39.2)	35.0 (30.9, 39.4)	30.2 (20.7, 41.6)	37.0 (30.0, 44.5)	40.9 (35.8, 46.2)	40.8 (32.4, 49.9)	38.3 (34.7, 42.1)	38.7 (28.5, 49.9)	45.8 (37.6, 54.2)
	Europe	■	■		■			■									30.9 (25.9, 36.4)	31 (26.8, 35.5)	29.6 (23.7, 36.3)	24.9 (16.3, 35.9)	41.8 (32.0, 52.4)	38.3 (32.5, 44.3)	38.4 (31.5, 45.9)	32.3 (25.6, 39.8)	31.7 (22.5, 42.6)	51.8 (40.0, 63.5)
	Americas			■	■	■					■						26.8 (23.2, 30.8)	26.1 (20.8, 32.2)	36.9 (33.3, 40.7)	25.6 (19.1, 33.5)	24.6 (17.9, 32.7)	34.0 (30.6, 37.6)	33.3 (27.1, 40.0)	40.1 (36.8, 43.4)	33.9 (28.0, 40.4)	30.2 (23.2, 38.2)
Hexavalent	Global	■	■	■	■	■		■									45.4 (39.0, 51.9)	47.0 (40.1, 54.0)	45.0 (38.9, 51.2)	39.4 (26.4, 54.2)	52.5 (41.9, 62.8)	56.6 (49.3, 63.6)	58.8 (47.7, 69.0)	48.9 (42.8, 55.0)	50.5 (36.5, 64.5)	65.7 (52.3, 77.0)
	Africa	■	■	■	■	■										■	43.8 (38.7, 49.0)	46.1 (38.8, 53.6)	41.9 (37.3, 46.5)	40.3 (30.3, 51.1)	47.8 (37.4, 58.3)	53.5 (48.6, 58.4)	56.6 (51.1, 62.0)	45.5 (41.4, 49.7)	51.2 (42.1, 60.3)	58.9 (47.3, 69.5)
	Asia		■	■	■	■		■								■	43.0 (37.9, 48.2)	41.3 (32.6, 50.5)	44.8 (41.1, 48.5)	43.2 (32.6, 54.6)	42.5 (37.7, 47.4)	53.5 (48.6, 58.2)	53.5 (44.2, 62.5)	48.4 (45.1, 51.8)	54.7 (45.3, 63.7)	53.8 (44.6, 62.8)
	Europe	■	■	■	■			■				■					42.4 (35.6, 49.5)	40.9 (35.6, 46.4)	38.7 (35.3, 42.2)	37.0 (22.0, 55.0)	55.8 (45.4, 65.7)	53.5 (45.3, 61.6)	49.5 (40.2, 58.8)	42.0 (38.7, 45.4)	46.6 (30.6, 63.4)	69.1 (56.9, 79.1)
	Americas		■	■	■	■		■			■						42.3 (37.1, 47.6)	38.1 (30.3, 46.5)	49.0 (44.6, 53.3)	41.3 (30.7, 52.7)	45.5 (37.8, 53.5)	52.7 (47.5, 57.8)	49.3 (39.3, 59.3)	53.3 (49.6, 56.9)	52.2 (42.6, 61.7)	57.1 (46.3, 67.3)
Octavalent	Global, Af, As	■	■	■	■	■		■					■			■	53.8 (47.7, 59.7)	53.5 (43.0, 63.6)	52.4 (48.3, 56.4)	54.1 (41.8, 66.0)	54.0 (43.9, 63.7)	66.9 (61.1, 72.2)	69.0 (59.3, 77.3)	56.8 (53.4, 60.1)	67.9 (56.9, 77.1)	67.6 (54.6, 78.4)
	Europe	■	■	■	■	■		■	■			■					50.9 (43.1, 58.5)	46.8 (39.1, 54.7)	46.9 (40.2, 53.8)	47.6 (29.3, 66.6)	63.4 (51.4, 73.9)	65.2 (56.4, 73.0)	60.3 (50.7, 69.2)	52.8 (43.5, 61.9)	59.9 (41.3, 76.1)	79.9 (64.7, 89.6)
	Americas		■	■	■	■		■		■	■				■		46.4 (40.8, 52.1)	41.4 (32.6, 50.9)	58.0 (51.1, 64.5)	45.4 (34.0, 57.3)	48.0 (41.5, 54.5)	57.7 (52.5, 62.7)	53.9 (43.2, 64.3)	63.3 (57.7, 68.6)	57.1 (47.3, 66.4)	59.9 (51.3, 68.0)
Decavalent	Global	■	■	■	■	■		■		■	■		■			■	60.1 (53.4, 66.4)	56.8 (46.3, 66.8)	64.9 (60.0, 69.5)	62.0 (48.1, 74.1)	59.0 (47.0, 70.1)	74.2 (68.6, 79.2)	73.2 (64.4, 80.6)	70.6 (66.9, 74.1)	76.7 (65.8, 84.9)	74.1 (59.5, 84.9)
	Africa	■	■	■	■	■		■		■			■	■		■	59.6 (52.6, 66.2)	59.4 (48.5, 69.4)	59.4 (54.6, 64.0)	60.8 (45.5, 74.2)	58.4 (47.3, 68.6)	73.9 (67.7, 79.3)	76.9 (66.9, 84.6)	64.4 (60.3, 68.3)	75.2 (62.4, 84.8)	72.7 (59.9, 82.7)
	Asia	■	■	■	■	■		■		■	■		■			■	60.1 (53.4, 66.4)	56.8 (46.3, 66.8)	64.9 (60.0, 69.5)	62.0 (48.1, 74.1)	59.0 (47.0, 70.1)	74.2 (68.6, 79.2)	73.2 (64.4, 80.6)	70.6 (66.9, 74.1)	76.7 (65.8, 84.9)	74.1 (59.5, 84.9)
	Europe	■	■	■	■	■	■	■	■	■		■					56.3 (48.3, 64.0)	49.7 (41.3, 58.2)	55.3 (47.5, 62.8)	54.1 (34.4, 72.6)	69.1 (57.8, 78.6)	71.9 (63.4, 79.1)	64.2 (54.1, 73.2)	61.8 (52.9, 70.0)	67.8 (48.6, 82.5)	86.0 (73.8, 93.0)
	Americas		■	■	■	■	■	■		■	■		■		■		53.8 (48.3, 59.2)	46.9 (37.4, 56.6)	65.7 (57.9, 72.8)	54.9 (44.1, 65.3)	52.0 (46.6, 57.3)	66.5 (61.9, 70.9)	61.1 (50.3, 71.0)	71.7 (65.4, 77.3)	68.3 (60.4, 75.2)	65.9 (58.7, 72.4)

Based on pooled prevalence estimates, different formulations for broadest possible coverage were assessed for each region (Af = Africa, As = Asia, E = Europe), as well as cumulatively (global). Shaded squares indicate inclusion of a capsule type in each respective formulation.
