# Supplementary material for: A systematic review and meta-analysis of Penner serotype prevalence of Campylobacter jejuni in low- and middle-income countries
Source: PLoS One. 2021 May 5;16(5):e0251039. doi: 10.1371/journal.pone.0251039 (PMC8099051; doi:10.1371/journal.pone.0251039)
Supplement: S1 Text — (PDF) [file pone.0251039.s019.pdf]

S1 Text. Search terms.

Database searches conducted on 19 July 2019.

PubMed:

("Campylobacter"[Mesh] OR "Campylobacter Infections"[Mesh] OR campylobacter*[tiab] OR jejuni[tiab] OR ((traveler*[tiab] OR traveller*[tiab]) AND (diarrhea*[tiab] OR diarrhoea*[tiab] OR diarhea*[tiab] OR diarhoea*[tiab] OR diahrrea*[tiab]))) AND ("Bacterial Capsules"[Mesh] OR "Capsules"[Mesh] OR "Serotyping"[Mesh] OR "Serogroup"[Mesh] OR serogroup*[tiab] OR "Polysaccharides, Bacterial"[Mesh] OR "Multiplex Polymerase Chain Reaction"[Mesh] OR Penner[all fields] OR serotyp*[tiab] OR serovar*[tiab] OR capsul*[tiab] OR encapsul*[tiab] OR heat stable[tiab] OR HS[tiab] OR HS1*[tiab] OR HS2*[tiab] OR HS3*[tiab] OR HS4*[tiab] OR HS5*[tiab] OR HS6*[tiab] OR HS7*[tiab] OR HS8*[tiab] OR HS9*[tiab] OR multiplex[tiab] OR polysaccharide*[tiab]) AND "English"[language]

Scopus:

TITLE-ABS-KEY(campylobacter* OR jejuni OR ((traveler* OR traveller*) AND (diarrhea* OR diarrhoea* OR diarhea* OR diarhoea* OR diahrrea*))) AND (TITLE-ABS-KEY(serogroup* OR serotyp* OR serovar* OR capsul* OR encapsul* OR "heat stable" OR HS* OR multiplex OR polysaccharide*) OR ALL(Penner)) AND (LIMIT-TO (LANGUAGE,"English"))

Web of Science Core Collection:

TS=(campylobacter* OR jejuni OR ((traveler* OR traveller*) AND (diarrhea* OR diarrhoea* OR diarhea* OR diarhoea* OR diahrrea*))) AND (TS=(serogroup* OR serotyp* OR serovar* OR capsul* OR encapsul* OR "heat stable" OR HS OR HS1* OR HS2* OR HS3* OR HS4* OR HS5* OR HS6* OR HS7* OR HS8* OR HS9* OR multiplex OR polysaccharide*) OR TS=(Penner))

Restrict to English
